# Predictive Value of Pre-Operative 2D and 3D Transthoracic Echocardiography in Patients Undergoing Mitral Valve Repair: Long Term Follow Up of Mitral Valve Regurgitation Recurrence and Heart Chamber Remodeling

**DOI:** 10.3390/jcdd7040046

**Published:** 2020-10-20

**Authors:** Gloria Tamborini, Valentina Mantegazza, Marco Penso, Manuela Muratori, Laura Fusini, Sarah Ghulam Ali, Claudia Cefalù, Gianpiero Italiano, Valentina Volpato, Paola Gripari, Enrico G. Caiani, Marco Zanobini, Mauro Pepi

**Affiliations:** 1 Centro Cardiologico Monzino, IRCCS, 20138 Milan, Italy; vmantegazza@ccfm.it (V.M.); mpenso@ccfm.it (M.P.); mmuratori@ccfm.it (M.M.); lfusini@ccfm.it (L.F.); sghulamali@ccfm.it (S.G.A.); ccefalu@ccfm.it (C.C.); gitaliano@ccfm.it (G.I.); vvolpato@ccfm.it (V.V.); pgripari@ccfm.it (P.G.); mzanobini@ccfm.it (M.Z.); mpepi@ccfm.it (M.P.); 2 Department of Electronics, Information and Bioengineering, Politecnico di Milano, 20133 Milan, Italy; enrico.caiani@polimi.it

**Keywords:** mitral valve prolapse, mitral valve repair, three-dimensional echocardiography, primary mitral regurgitation, mitral valve disease

## Abstract

The “ideal” management of asymptomatic severe mitral regurgitation (MR) in valve prolapse (MVP) is still debated. The aims of this study were to identify pre-operatory parameters predictive of residual MR and of early and long-term favorable remodeling after MVP repair. We included 295 patients who underwent MV repair for MVP with pre-operatory two- and three-dimensional transthoracic echocardiography (2DTTE and 3DTTE) and 6-months (6M) and 3-years (3Y) follow-up 2DTTE. MVP was classified by 3DTTE as simple or complex and surgical procedures as simple or complex. Pre-operative echo parameters were compared to post-operative values at 6M and 3Y. Patients were divided into Group 1 (6M-MR < 2) and Group 2 (6M-MR ≥ 2), and predictors of MR ≥ 2 were investigated. MVP was simple in 178/295 pts, and 94% underwent simple procedures, while in only 42/117 (36%) of complex MVP a simple procedure was performed. A significant relation among prolapse anatomy, surgical procedures and residual MR was found. Post-operative MR ≥ 2 was present in 9.8%: complex MVP undergoing complex procedures had twice the percentage of MR ≥ 2 vs. simple MVP and simple procedures. MVP complexity resulted independent predictor of 6M-MR ≥ 2. Favorable cardiac remodeling, initially found in all cases, was maintained only in MR < 2 at 3Y. Pre-operative 3DTTE MVP morphology identifies pts undergoing simple or complex procedures predicting MR recurrence and favorable cardiac remodeling.

## 1. Introduction

Myxomatous or degenerative mitral valve (MV) disease is the leading cause of MV prolapse (MVP) and surgically correctable mitral regurgitation (MR) in the developed world. International guidelines suggest early MV repair prior to the development of symptoms or left ventricular (LV) dysfunction [1]. However, the correct timing of MV repair is still under debate, and discordance between observational investigations of watchful waiting and early surgery management strategies drives the continued controversy surrounding this issue [2,3,4,5]. In this regard, recent guidelines stated that MV repair can be considered in asymptomatic patients when there is a high likelihood of durable result at very low risk (<1% mortality). Results in terms of reparability and recurrence of MR are related to clinical (younger age) and surgical factors (simple versus complex surgical procedures) [6,7,8,9] and varies in different studies. Since more complex surgical procedures are mainly indicated in cases with more complex prolapses and vice versa, noninvasive pre-operative assessment of MV anatomy is essential to define feasibility and complexity of repair. Moreover, three-dimensional (3D) transthoracic echocardiography (TTE) is nowadays a very robust technique that allows a very precise localization and definition of MV pathology and a comprehensive two-dimensional (2D) TTE, and 3DTTE evaluation may, therefore, include all morphologic, hemodynamic and functional data [10,11,12,13,14]. The aims of this study in a large series of patient undergoing MV repair for severe MVP were: (a) to evaluate whether 3DTTE may identify cases undergoing simple vs. complex surgical procedures based on the MVP complexity; (b) to correlate MVP complexity and surgical techniques to outcomes in terms of 6 months (6M) and 3 years (3Y) MR residual severity; (c) to correlate all these findings (mainly optimal results vs. recurrence of MR) to left chamber remodeling and functional and hemodynamic parameters at 6M and 3Y follow-up.

## 2. Methods

Between 2008 and 2018, 1000 cases with severe MR due to MVP underwent early MV repair in Centro Cardiologico Monzino IRCCS. We retrospectively selected all consecutive cases with a pre-operative 2DTTE and a 3DTTE as well as a 2DTTE at 6M and 3Y follow-up. Of the 1000 cases, 13 patients were excluded for an insufficient quality of 3DTTE reconstruction, 541 patients because after MV repair the requested follow-up was not available and 51 cases because they underwent MV replacement after a first attempt of repair. Thus, our final study population was represented by 295 patients. The local research committee approved this retrospective study protocol (reference N° R1262/20-CCM 1326) and all study participants provided written informed consent.

All the 295 patients in the study population underwent a 2DTTE and 3DTTE within 1 month prior to surgery. Two-dimensional and 3DTTE were performed using 2 ultrasound platforms (iE33 or EPIQ 7C) both equipped with the X5-1 probe (Philips Medical Systems, Andover, MA, USA).

From 2DTTE, we derived left ventricular (LV) end-diastolic (EDVI) and end-systolic (ESVI) volumes indexed for body surface area (BSA); LV ejection fraction (EF), LV stroke volume (SV), left atrial volume indexed for BSA (LAVI) using the biplane Simpson’s method. Grading of tricuspid regurgitation (mild = 1, moderate = 2, severe = 3) was obtained according to guidelines [15]. Pulmonary artery systolic pressure (PASP) was calculated using the Doppler echocardiographic method [16].

All pre-op 2DTTE and 3DTTE images were retrospectively analyzed by a single experienced echocardiographer, blinded to the intraoperative findings. The Carpentier nomenclature was applied to the MV leaflets [16]. The presence of ruptured chordae, clefts, annular or leaflet severe calcifications was annotated. MVP was defined as simple or complex: simple anatomical lesions included isolated P2 prolapse or P2 associated with P1 or P3. According to literature [3,17,18,19,20], cases including lesions involving >2 posterior leaflet scallops, anterior or both leaflets, commissures or with severe annular or leaflet calcifications were defined as complex. The 2 main phenotypes of MVP were also distinguished: Barlow disease (mixomatous leaflet degeneration, elongated and thickened chordae, dilated annulus) and fibroelastic deficiency (FED) (normal/thinner leaflets, frequent single segment prolapse with chordal rupture) [9,17,18,19].

To assess reproducibility of MVP evaluation (prolapsing scallop identification), intraobserver variability was performed by the same reader after ≥1 month; interobserver variability was performed by a second experienced reader, blinded to the previous results

Echocardiographic MVP evaluation was compared with MV anatomical inspection performed by the operating surgeon.

Protocols and reports of surgical techniques were annotated in detail. According to literature data [20,21,22] and surgical institutional experience, surgical procedures were divided into simple vs. complex techniques. Recently, the complexity of surgical procedures was better defined [23,24].

Other associated procedures were recorded.

A score of 1 (mild), 2 (mild-to-moderate), 3 (moderate-to-severe) or 4 (severe) was assigned to MR integrating both qualitative and quantitative parameters [15,25]. Based on 6M-MR, patients were divided into Group 1 (residual MR < 2) and Group 2 (residual MR ≥ 2). Differences in left chamber volumes and functional parameters, pre-op MVP morphology (complex vs. simple) and surgical procedure (complex vs. simple) were compared between the 2 groups and analyzed to identify outcome predictors.

Continuous data are reported as the mean ±standard deviations, whereas categorical data as absolute frequencies (percentages). A test of normality (Shapiro-Wilk test) was performed on continuous data. Continuous variables were compared using the unpaired Student’s *t*-test (and the Welch’s corrected version, as appropriate) or the Mann-Whitney U test, whilst a χ^2^ test was applied for categorical data. Repeated measures one-way ANOVA test or Friedman test with the Bonferroni correction were used to evaluate TTE mean values at baseline, 6M and 3Y. Inter- and intraobserver correlations were performed using Pearson coefficient. Kruskal Wallis and Mann–Whitney U test with Bonferroni correction was used to compare residual mitral regurgitation in each group of patients according to MVP anatomy and surgery technique All results were considered significant with *p*-value < 0.05. Echocardiographic parameters as well as baseline prolapse and procedure characteristics with a *p*-value < 0.10 at univariate logistic analysis were used for the multivariate logistic regression analysis with the enter method for the identification of independent variables associated with the outcome. All the statistical analyses were implemented using IBM SPSS 25.

## 3. Results

Patient characteristics data in the whole study population and in each group of patients are shown in Table 1.

Age (58 ± 14 vs. 61 ± 11, *p* < 0.05) and BSA (1.78 ± 0.19 vs. 1.84 ± 0.14 cm^2^, *p* < 0.05) were significantly lower in complex in comparison with simple MVP. A complex MVP was more frequent in Barlow’s disease patients (98/224, 43.7%) in comparison with FED (19/71, 26.7%). There was close agreement in echocardiographic MVP assessment both between the 2 different observers (interobserver variability: r = 0.88, *p* < 0.001), and between the repeated measurement of the same observer (intraobserver variability: r = 0.96 *p* < 0.001). Surgical inspection confirmed echocardiographic diagnosis of the prolapsing scallops in 292/295 cases (98.9%). In 3 cases without agreement between surgical inspection and echocardiographic diagnosis, the anatomical findings did not change MVP classification (simple or complex). Table 2 shows surgical techniques for MV repair and associated procedures performed.

**Figure 1:**
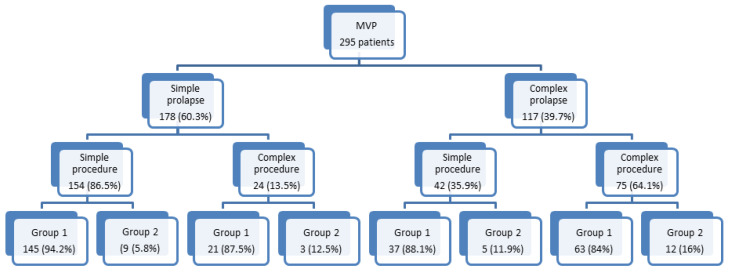
Flow-chart scheme showing the number and percentage of cases with simple vs. complex mitral valve lesions, types of cardiac surgery according to complexity of mitral lesions and 6 month residual mitral valve regurgitation ≤2 (Group1) or >2 (Group 2). MVP = mitral valve prolapse.

A simple surgical procedure was realized in most of simple MVPs; conversely, complex procedures were performed in the majority of complex prolapses (Figure 1).

Baseline, 6M and 3Y 2DTTE parameters are summarized in Table 3.

Residual MR increased significantly from 6M to 3Y both in patients with simple MVP and with complex prolapse. However, at 3Y, patients with simple MVP and simple surgery had alower increase in comparison with cases with complex MVP and complex surgery (Table 4)

After surgery reduction in LVEDVI, LVESVI and left atrial volumes were observed at 6M. The expected reduction in MR after MV repair was also associated with a reduction in LVSVI, LVEF and PASP. At 3Y no additional changes were found in LV and left atrial parameters, while a mild but significant increase in PASP was observed. Based on 6M residual MR, 266 patients were included in Group 1 (6M-MR < 2) and 29 in Group 2 (6M-MR ≥ 2). No differences in gender, age, BSA or incidence of atrial fibrillation were observed between the 2 groups. (Table 1). No significant differences in pre-operative echo data were present between Group 1 and Group 2 with the exception of PASP which was significantly higher in Group 2.

At 6M, changes in left cardiac chambers and PASP were similar to the all population although reduction in LVESVI did not reach statistical significance in Group 2. At 3Y, both groups showed marked differences both in MR severity, chambers remodeling and PASP. Indeed, in Group 1 no differences were observed in LV volumes or PASP at 3Y vs. 6M data. On the contrary, a small but significant decrease in LAVI and an improvement in LVEF was present. In Group 2, a trend towards larger left chambers volumes and lower LVEF was observed at 3Y vs. 6M, associated with a significant increase in PASP. Residual MR ≥ 2 was significantly more frequent in complex MVP in comparison with simple cases and, as expected, after complex surgery than after a simple procedure.

X^2^ analysis demonstrated a significant relationship between prolapse anatomy, type of procedure and residual 6M-MR. The percentage of patients with complex MVP undergoing complex procedure with residual MR ≥ 2 is more than twice than the percentage of patients with simple prolapse undergoing simple procedures (5.8% vs. 16%). At univariate analysis PASP, LVSVI, MVP morphology (simple vs. complex) and procedure (simple vs. complex) were the only independent predictors of MR ≥ 2 (Table 5).

We identified from the baseline parameters the complex MVP, complex procedure, PASP as independent predictors of the MR at 6M. At multivariate analysis, MVP complexity and PASP were found as independent predictors of 6M-MR ≥ 2.

**Figure 2:**
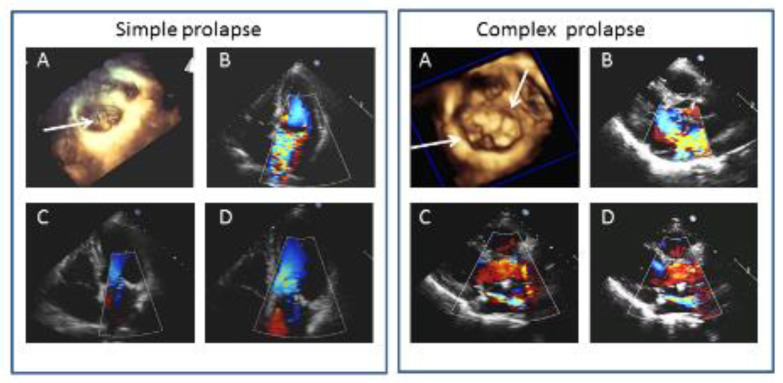
Left panels: Example of simple mitral valve prolapse (MVP). (**A**) surgical view of three-dimensional MVP reconstruction showing P2 prolapse with multiple chordal ruptures (arrow). (**B**) two-dimensional transthoracic 4 chamber apical view showing a severe mitral regurgitation (MR). (**C**,**D**) two-dimensional transthoracic 4 chamber apical views at 6M and 3Y follow-up, respectively, with trivial MR. Right panels: example of complex MVP. (**A**) surgical view of three-dimensional MVP reconstruction showing A2, A3, P2 prolapse (arrows) (**B**) two-dimensional transthoracic parasternal view showing severe MR central jet. (**C**,**D**) two-dimensional parasternal long axis views at 6M and 3Y follow-up, respectively, with mild to moderate MR.

Figure 2 shows 2 examples of MVP before surgery and at 6M and 3Y.

## 4. Discussion

The mean findings of this study are (a) 3DTTE may facilitate the evaluation of MV morphology in patients with MVP undergoing surgical repair; (b) a comprehensive 2DTTE and 3DTTE allowed to discriminate simple vs. complex lesions and to facilitate the prediction of surgical procedures. In cases with simple lesions undergoing simple procedures, the absence of significant MR at follow-up correlated with favorable LV and left atrial remodeling and normalization of PASP. Recurrence of MR ≥ 2 at 3Y occurred in a small subgroup of patients undergoing complex procedures for complex lesions, and in these cases, after an initial favorable LV and left atrial remodeling at 6M a trend towards larger left chamber volumes and lower LVEF as well as a significant increase in PASP was observed at 3Y. Thus, pre-operative 2DTTE and 3DTTE may facilitate the prediction of surgical complexity, and results in terms of MR recurrence and heart remodeling.

Three-dimensional TTE may be performed routinely as previously demonstrated [10,11,12,13,14] and an accurate assessment of MV morphology may be easily obtained. Indeed, even though 3DTEE provides MV images of extraordinary quality with a very high accuracy in the identification of all scallops, head-to-head comparison among 2D-3DTTE and 2D-3DTEE confirmed previous data suggesting that besides overall 3DTEE accuracy being the highest over every other echocardiographic techniques, 3DTTE accuracy is similar to 2DTEE (central scallops) or slightly superior (lateral and medial scallops) than 2DTEE [10]. Therefore, a comprehensive transthoracic approach may include not only all standard echo-Doppler measurements but also 3D morphology of the MV. This may be particularly useful in the follow-up of patients avoiding TEE studies or performing TEE studies only when TTE is suboptimal or when specific MV details have to be further investigated before surgery.

Moreover, it has been previously demonstrated that 3DTTE is feasible, timesaving and accurate in identifying simple vs. complex MVP [12]. Recently new advantages in 3D technology further facilitated visualization of the MV. Imaging quality is also improved, and good or optimal quality was found in the majority of cases confirming also a 95% overall accuracy of 3DTTE in the recognition of MV lesions. This percentage was obtained in this large (295 patients) unselected population—a percentage similar to previous 2DTTE and 3DTTE studies [10,11,12].

Early surgery prior to the development of symptoms and LV dysfunction is recommended independently on MV complexity. Javadikasgari et al. recently demonstrated that valve repair was associated with similarly low operative risk- and time-related survival but less durability in complex disease [3]. Indeed, several studies showed that patients with simple and complex degenerative MR undergoing MV repair, despite similar low operative risk may have different long-term outcomes [4,5,6,7,8]. Earlier operation and placing artificial chordae in complex MV disease and not having leaflet resection and annuloplasty were associated with lower durability of MV repair. In the last two decades, patients have been referred for early correction of severe MR regardless of the type and extent of MVP. In a recent review, David [5] stated that MV repair for degenerative MR is associated with a low probability of reoperation for up to two decades after surgery. However, almost one-third of the patients develop recurrent moderate or severe MR suggesting that surgery does not arrest the degenerative process. [6]. Other anatomical characteristics may also affect surgical outcomes, and Fusini [26] showed that mitral annular calcification is a relative common finding in MVP and demonstrated in a series of 410 patients that 8% of cases with annular calcifications underwent replacement after a first attempt of repair vs. 3% without. For all these reasons, the correct timing of asymptomatic patients with severe MR is still under debate, and guidelines [1] not only state that MV repair can be considered when there is a high likelihood of durable MV repair at low risk but also introduced detailed characteristics of the ideal candidate for early surgery.

In our study, we sought to demonstrate that a comprehensive 2D and 3DTTE may orient to the correct timing of surgery and may predict cases with a lower probability of durable MV repair. The large majority of patients in our series had excellent early and long-term results and only 29 out of 295 patients (9%) had a MR ≥ 2 at 3Y. Interestingly, MVP complexity as well as procedure complexity correlated to outcomes. The number of cases with MR ≥ 2 at 3Y that have performed a complex procedure was more than twice than the percentage of simple prolapse and simple procedures with MR ≥ 2 (16% vs. 5.8%). Moreover, cases with more complex MVP and suboptimal results also had other pre-operative characteristics such as higher grade of tricuspid regurgitation and higher PASP. At multivariate analysis, only MVP anatomy and baseline PASP were independent predictors of residual MR.

To prevent irreversible LV dysfunction that may occur despite an apparently “normal” LV with a preserved LVEF, guidelines suggest early MV repair (when feasible) in asymptomatic patients with preserved LV systolic function. This strategy not only avoids the appearance of symptoms (in the “old” strategy of waiting for symptoms, MV repair was associated with the worst prognosis, in terms of post-operative mortality and risk of heart failure) but allows a favorable LV and left atrial remodeling [27,28,29]. Novelties of this study are that the remodeling of LV, left atrium and changes in hemodynamic status correlated to the pre-operative comprehensive echo-Doppler values, to the MVP characteristics and to surgical results in terms of residual MR both at early post-operative observation and at long term follow-up.

We selected consecutive asymptomatic patients undergoing MV repair who had a complete clinical and echo-Doppler follow-up. All cases had favorable remodeling of LAVI and LVEDVI, associated with a mild decrease in LVEF and reduction in PASP. However, patients with residual MR ≥ 2 differed significantly in terms of early and mainly late remodeling and hemodynamics. At 3Y, LAVI and LV volumes were steadily reduced in patients with optimal MV repair results (MR < 2), while the minority of cases with suboptimal results showed an initial favorable remodeling that did not persist at 3Y. Accordingly, PASP significantly decreased in both groups in the early post-operative period, but only in cases with suboptimal repair it significantly increased thereafter.

Suri [30] demonstrated that early repair of MVP, before deterioration in left heart size or function, increases the likelihood of subsequent normalization of LVEF. Our data confirmed favorable remodeling of left heart chambers associated with values of LVEF within the range of normality throughout the follow-up. Interestingly early mild decrease in LVEF after repair (as expected due to increased afterload) and tendency to increase values at 3Y occurred in Group 1, whereas LVEF was steadily reduced at 3Y in comparison to pre-operative values (even though in the range of normal values) in Group 2.

Our data finally support the role of 3DTTE not only in patients in patients in whom the surgical indication is obvious, but also in the pre-operative follow-up of patients without surgical indication in whom serial examinations are very useful to determine the correct surgical timing. Therefore, a comprehensive 2D and 3DTTE may reduce the need for a TEE approach. Indeed, our data showed that the overwhelming majority of cases with simple lesions underwent a simple surgical technique, while patients with complex prolapse could be treated by simple or complex successful MV repair. Moreover, the recurrence of MR ≥ 2 at 6M and the MR increase at 3Y are higher in cases undergoing complex procedures for complex lesions.

## 5. Conclusions

The complexity of MV repair procedure may be predicted by a comprehensive 2DTTE and 3DTTE, thus facilitating the correct timing of surgery (early vs. late procedure). Finally, correct timing in cases with optimal early and late results is related to normalization of left heart chamber volumes, maintenance of normal LVEF and normalization of PASP, while after an initial favorable LV and LAVI remodeling at 6M, a trend towards larger left chamber volumes and lower LVEF was observed associated with a significant increase in PASP at 3Y in cases with residual significant MR.

## Figures and Tables

**Table 1:** Mean ±SD and/or frequencies of baseline clinical characteristics of the study population.

	Overall Population (*n* = 295)	Group 1: 6M-MR < 2 (*n* = 266)	Group 2: 6M-MR ≥ 2 (*n*= 29)	*p* Value
Age (years)	60 ± 13	60 ± 12	63 ± 15	0.116
Men	197 (66.8%)	182 (68.4%)	15 (51.7%)	0.070
Body surface area (m^2^)	1.8 ± 0.2	1.8 ± 0.2	1.8 ± 0.2	0.084
Atrial fibrillation	24 (8.1%)	22 (8.3%)		0.797
Etiology				0.654
FED	71 (24.1%)	65 (24.4%)	6 (20.7%)	
Barlow	224 (75.9%)	201 (75.6%)	23 (79.3%)	
Prolapse anatomy				0.028
Simple	178 (60.3%)	166 (62.4%)	12 (41.4%)	
Complex	117 (39.7%)	100 (37.6%)	17 (58.6%)	
Type of procedure				0.029
Simple	196 (66.4%)	182 (68.4%)	14 (48.3%)	
Complex	99 (33.6%)	84 (31.6%)	15 (51.7%)	

6M-MR: residual mitral regurgitation at 6-month follow-up.

**Table 2:** Surgical techniques used in the study population.

Simple Surgical Techniques for Mitral Valve Repair	
Quadrangular resection of posterior leaflet	292 (98.9%)
Annuloplasty	294 (99.6%)
Sliding of posterior leaflet/Posterior leaflet folding plasty	112 (38%)
Edge-to-edge technique	30 (1%)
Posterior annular plication	88 (29.3%)
Commissural fusion or Cleft Closure	28 (9.4%)
**Complex Surgical Techniques for Mitral Valve Repair**	
Replacement or transfer of chordae tendinae	71 (24%)
Papillary muscle repositioning	44 (14.9%)
**Other Associated Procedures**	
Aorto-coronary bypass graft	31 (10%)
Tricuspid valve annuloplasty	48 (16.2%)
Aortic valve/root replacement	23 (7.8%)
Atrial fibrillation ablation	23 (7.8%)
Atrial appendage closure	17 (5.7%)
Patent forame ovale closure	7 (2.3%)

**Table 3:** Mean ±SD for 2D transthoracic echocardiographic parameters at baseline and at 6 months and 3 years follow-up.

	Basal	6 Months	3 Years	*p*-Value
All Patients (295 patients)				
Mitral regurgitation grade	3.9 ± 0.2	0.6 ± 0.7 *	0.9 ± 0.9 ^†,^*	<0.001
Left ventricular end diastolic volume index (mL/m^2^)	77.4 ± 19.3	57.3 ± 14.9 *	56.9 ± 17.8 *	<0.001
Left ventricular end systolic volume index (mL/m^2^)	27.0 ± 9.6	24.6 ± 10.7 *	23.9 ± 12.3 *	<0.001
Left ventricular stroke volume index (mL/mq)	50.4 ± 12.8	32.6 ± 7.5 *	32.9 ± 8.3 *	<0.001
Left ventricular ejection fraction (%)	65.2 ± 6.8	57.9 ± 8.0 *	59.2 ± 7.7 ^†,^*	<0.001
Left atrial volume index (mL/m^2^)	66.4 ± 24.8	45.4 ± 17.7 *	44.5 ± 18.5 *	<0.001
Tricuspid regurgitation grade	1.0 ± 0.8	0.9 ± 0.4	1.0 ± 0.4 ^†^	0.024
Pulmonary artery systolic pressure (mmHg)	36.4 ± 11.1	28.1 ± 5.9 *	29.5 ± 7.6 ^†,^*	<0.001
Group 1: 6M-MR < 2 (266 patients)				
Mitral regurgitation grade	3.9 ± 0.2	0.4 ± 0.4 *	0.7 ± 0.7 *^,†^	<0.001
Left ventricular end diastolic volume index (mL/m^2^)	77.0 ± 18.9	56.7 ± 14.6 *	55.9 ± 17.0 *	<0.001
Left ventricular end systolic volume index (mL/m^2^)	26.8 ± 9.2	24.5 ± 10.7 *	23.4 ± 11.9 *	<0.001
Left ventricular stroke volume index (mL/m^2^)	50.1 ± 12.7	32.2 ± 7.2 *	32.5 ± 8.0 *	<0.001
Left ventricular ejection fraction (%)	65.2 ± 6.7	57.7 ± 7.8 *	59.3 ± 7.6 ^†,^*	<0.001
Left atrial volume index (mL/m^2^)	66.1 ± 24.4	44.5 ± 17.5 *	43.1 ± 17.7 ^†,^*	<0.001
Tricuspid regurgitation grade	1.0 ± 0.8	0.9 ± 0.3	1.0 ± 0.4	0.066
Pulmonary artery systolic pressure (mmHg)	35.4 ± 9.8	27.7 ± 5.6 ^*^	28.7 ± 6.8 *	<0.001
Group 2: 6M-MR ≥2 (29 patients)				
Mitral regurgitation grade	3.9 ± 0.3	2.1 ± 0.3 ^‡,^*	2.7 ± 0.7 ^‡,†,^*	<0.001
Left ventricular end diastolic volume index (mL/m^2^)	82.2 ± 22.8	62.9 ± 16.0 ^‡,^*	66.0 ± 22.0 ^‡,^*	<0.001
Left ventricular end systolic volume index (mL/m^2^)	29.4 ± 12.7	25.9 ± 10.7	28.8 ± 14.7 ^‡^	0.263
Left ventricular stroke volume index (mL/m^2^)	52.7 ± 13.6	37.0 ± 8.8 ^‡,^*	37.1 ± 9.6 ^‡,^*	<0.001
Left ventricular ejection fraction (%)	65.1 ± 7.7	59.8 ± 9.6 *	57.8 ± 8.2 *	<0.001
Left atrial volume index (mL/m^2^)	68.9 ± 28.1	53.6 ± 17.6 ^‡,^*	56.6 ± 21.8 ^‡,^*	0.002
Tricuspid regurgitation grade	1.3 ± 1.0	1.1 ± 0.7	1.3 ± 0.7 ^‡^	0.270
Pulmonary artery systolic pressure (mmHg)	45.2 ± 16.8 ^‡^	31.8 ± 7.4 ^‡,^*	37.0 ± 10.1 ^‡,†,^*	<0.001

* *p* < 0.05: vs. baseline, ^†^ *p* < 0.05: vs. 6 months, ^‡^ *p* < 0.05: mitral regurgitation ≥2 vs. <2.

**Table 4:** Six-months and 3-years residual mitral regurgitation according to MVP anatomy and surgery technique reported as median (25th–75th percentile).

	6 Months	3 Years	*p*-Value
Simple MVP + simple surgery	0.50 (0.00–1.00)	0.75 (0.00–1.00) *	<0.001
Simple MVP + complex surgery	0.25 (0.00–1.00)	0.75 (0.12–1.37) *	0.003
Complex MVP + simple surgery	0.50 (0.00–1.00)	1.00 (0.50–1.00) *	0.002
Complex MVP + complex surgery	0.50 (0.00–1.00)	1.00 (0.50–2.00) *^,#^	<0.001

* *p* < 0.05, 6 months vs. 3 years; ^#^ *p* < 0.05 significant difference in increase in MR severity among groups.

**Table 5:** Results of univariate and multivariate analysis for the identification of independent variables predicting residual mitral regurgitation ≥2.

	Univariate Model		Multivariate Model	
	OR (95% CI)	*p* Value	OR (95% CI)	*p* Value
Male	0.747 (0.396–1.408)	0.367		
Age (years)	1.009 (0.984–1.034)	0.490		
Body surface area (m^2^)	0.984 (0.204–4.754)	0.984		
Antero-posterior mitral anulus diameter (mm)	1.009 (0.951–1.070)	0.774		
Medio-lateral mitral anulus diameter (mm)	0.996 (0.935–1.061)	0.906		
Left ventricular stroke volume index (mL/mq)	1.020 (0.998–1.044)	0.081	1.018 (0.993–1.043)	0.144
Left ventricular ejection fraction (%)	0.989 (0.945–1.034)	0.628		
Left atrial volume index (mL/mq)	1.005 (0.994–1.017)	0.377		
Tricuspid regurgitation ≥ 2	0.619 (0.303–1.262)	0.187		
Systolic pulmonary pressure (mmHg)	1.054 (1.028–1.082)	<0.001	1.056 (1.029–1.084)	<0.001
Etiology (Presence of fibroelastic deficiency)	1.764 (0.785–3.965)	0.169		
Complex mitral valve prolapse	0.425 (0.228–0.792)	0.007	0.414 (0.215–0.798)	0.008
Complex surgical procedure	0.457 (0.245–0.851)	0.014	0.646 (0.297–1.407)	0.247
